# Changing the organizational work schedule of shift workers leads to improved sleep—an editorial

**DOI:** 10.1093/sleep/zsae103

**Published:** 2024-04-29

**Authors:** Annie Vallières, Megan R Crawford

**Affiliations:** École de psychologie, Université Laval, Québec, QC, Canada; Centre de recherche CERVO, Québec, QC, Canada; Centre de recherche du CHU de Québec-Université Laval, Québec, QC, Canada; Strathclyde Centre for Sleep Health, Department of Psychological Sciences and Health, University of Strathclyde, Glasgow, UK

Shift work is defined as working outside the conventional daytime hours of 6 am to 6 pm. Shift work can also be called atypical work schedules or nonstandard work schedules. As many as 20% to 29% of workers worldwide engage in shift working of some type [1]. It can take many different forms including night shift, evening shift, on-call shift, long working hours, and quick returns, to name a few. As diurnal species, humans are naturally adapted to daytime activity and nighttime rest, making shift working conditions inherently unnatural. Research has consistently shown the significant impact these conditions can have on sleep and overall health; however, the extent of this impact varies depending on the type of shift work [2]. Quick returns as one type of shift work may be particularly problematic. Quick returns are defined by two shifts following on from each other, with less than 11 hours in between. This occurs often between an evening shift and an early morning or a day shift. Although not permitted in every country, quick returns can also result from unplanned prolonged shift durations, whether by choice or required, leading to a shortened interval between the current shift and the next shift, resulting in a quick return. The prolonged shift may happen for diverse reasons such as cover for staff on sick leave or unplanned shift swaps.

Quick returns are known to reduce sleep and increase sleepiness and fatigue [3]. Moreover, experiencing quick returns increases the probability of experiencing insomnia [4]. To understand the link between quick returns and insomnia, it is important to note that workers on quick returns have only 11 hours (or less) between the two shifts to manage commuting time between work and home, meal preparation, family management (if applicable), physical activity, and sleep. As a result, due to this time constraint, workers may experience an urge to sleep, leading to increased anxiety about falling asleep and, consequently, insomnia. Additionally, workers may choose to sacrifice sleep in order to accommodate all other activities within the limited timeframe between shifts. These possibilities can result in either sleep deprivation or insomnia, both of which are known to have severe and complex consequences on physical and mental health [5, 6].

Considering the significant impact of shift work on health and well-being, and the large portion of the population that engages in shift work, it is paramount that we develop interventions to improve the conditions of shift workers with the goal of reducing fatigue, sleepiness, insomnia, and improving health [7]. Any efforts to do this *without* taking the multifaceted complexities of shift work into consideration will likely fail. Individual, occupational, and sociological factors are involved in shift work and need to be considered in any intervention aiming to improve shift workers’ health and well-being, as well as work satisfaction, productivity, and decreasing work errors/accidents and related costs. This requires close collaboration among all stakeholders: the researchers, the workers, and the workplace. The research conducted from this perspective is referred to as participatory research [8] and falls within the framework of an integrated knowledge translation approach [8]. The collaboration between the researchers and the workplace facilitates the direct translation of applicable knowledge into the health system, ultimately benefiting society [8]. The result of the collaboration will be an effective intervention that is implemented in the setting in real-time. Conducting participatory research within an integrated knowledge translation approach while maintaining scientific rigor is challenging. However, Djupedal et al. [9] have successfully met the challenge. They used a rigorous and well-controlled randomized trial design, while simultaneously collaborating with the relevant stakeholders to implement an intervention in the intended setting. This serves as an excellent demonstration of participatory research in action within the field of sleep and shift work.

The results are quite clear: the abated quick return intervention reduced insomnia symptoms and sleepiness, with small effect sizes. It has to be highlighted that the study did not target workers with a sleep disorder diagnosis but rather a population of workers that may include a range of insomnia and sleepiness difficulties from none to severe. In that context, to observe an impact on sleep by changing the work schedules is even more meaningful. Often sleep is not considered as an outcome to target because it is perceived as the individual worker’s “problem” to solve rather than something the organization can have an impact on. On the contrary, Djupedal et al. demonstrate that sleep can be successfully improved from an organizational perspective, after direct involvement from the employer (through changes in the work schedules).

Djupedal et al. [9]’s study possesses several strengths worth naming. First, they explicitly prioritize insomnia as their primary outcome, affirming its high prevalence among shift workers [10, 11]. Second, the sample size is large and within the same hospital, providing high statistical power. Third, the hospital registry is utilized to record adherence to the work schedules and frequency of quick returns, thereby offering robust data on the work schedules themselves. Four, hospital units were matched, and the study controlled for seasonal effects, which could have biased the results because of light-related issues associated with seasons that may affect sleep. However, the response rate, which is a bit low, is a limitation acknowledged by the authors. It is worth noting that the response rate may reflect both the employee mobility within the hospital and the difficulties in implementing such interventions in the workplace.

Interestingly the authors also outline some of the unintended side effects. Some workers reported that having fewer quick returns complicated the family-work balance rather than facilitating it. Other workers reported that the continuity of care for patients was more complicated with fewer quick returns. These align with another study on shift work showing that healtcare night shift workers tend to skip meals at work for the benefit of the patients’s care [12] and may thus reflect the healthcare workers’ representations of their work. It may also be a reflection that getting good sleep is not a priority for some of these individuals and is sacrificed to perform better at work. Whether they really do perform better at work on less sleep, or misjudge the negative impact the lack of sleep has on cognitive functioning needs to be determined. Future studies should also include objective measures of performance in order to establish the impact of abated quick returns on functioning. Nevertheless, negative effects (whether real or misperceived) could be barriers to successful implementation in other hospitals or settings. Further investigation into work schedule arrangements might include evaluating family-work balance, as well as nature of work and its representation, to address potential implementation barriers.

For the future, the study of shift workers' sleep will benefit from participatory research studies such as Djupedal et al. [9]. Furthermore, improving sleep in shift workers will come through the consideration of the complexities of shift work. Individual interventions targeting sleepiness, fatigue, and/or insomnia need to be successfully adapted to shift work [13, 14]. Sociological factors involved in shift work that make shift workers sacrifice their sleep opportunities have also to be identified to raise awareness about sleep importance. Occupational factors, beautifully addressed by Djupedal et al. [9], need to be replicated and extended to other work schedules. Ultimately, the future of improving sleep in shift work likely lies in a combination of interventions finely tailored to accommodate the diverse array of work schedule possibilities under the shift work umbrella.

## References

[1] IARC Monographs Vol 124 group (2019). Carcinogenicity of night shift work. Lancet Oncol. 2019;20(8):1058–1059. doi: 10.1016/S1470-2045(19)30455-3 31281097

[2] Zhao Y , Richardson A, Poyser C, Butterworth P, Strazdins L, Leach LS. Shift work and mental health: a systematic review and meta-analysis. Int Arch Occup Environ Health. 2019;92(6):763–793. doi: 10.1007/s00420-019-01434-3 31055776

[3] Vedaa O , Harris A, Erevik EK, et al. Short rest between shifts (quick returns) and night work is associated with work-related accidents. Int Arch Occup Environ Health. 2019;92(6):829–835. doi: 10.1007/s00420-019-01421-8 30879132

[4] Eldevik MF , Flo E, Moen BE, Pallesen S, Bjorvatn B. Insomnia, excessive sleepiness, excessive fatigue, anxiety, depression and shift work disorder in nurses having less than 11 hours in-between shifts. PLoS One. 2013;8(8):e70882. doi: 10.1371/journal.pone.0070882 23976964 PMC3744484

[5] Medic G , Wille M, Hemels ME. Short- and long-term health consequences of sleep disruption. Nat Sci Sleep. 2017;9:151–161. doi: 10.2147/NSS.S134864 28579842 PMC5449130

[6] Wu TT , Zou YL, Xu KD, et al. Insomnia and multiple health outcomes: umbrella review of meta-analyses of prospective cohort studies. Public Health. 2023;215:66–74. doi: 10.1016/j.puhe.2022.11.021 36645961

[7] Reynolds AC , Lechat B, Melaku YA, et al. Shift work, clinically significant sleep disorders and mental health in a representative, cross-sectional sample of young working adults. Sci Rep. 2022;12(1):16255. doi: 10.1038/s41598-022-20308-2 36171220 PMC9519578

[8] Jull J , Giles A, Graham ID. Community-based participatory research and integrated knowledge translation: advancing the co-creation of knowledge. Implement Sci. 2017;12(1):150. doi: 10.1186/s13012-017-0696-3 29258551 PMC5735911

[9] Djupedal ILR , Harris A, Svensen E, et al. Effects of a work schedule with abated quick returns on insomnia, sleepiness and work-related fatigue: results from a large-scale cluster randomized controlled trial. Sleep. 2024;47(7):zsae086. doi: 10.1093/sleep/zsae086 PMC11236942 38581363

[10] Brito RS , Dias C, Afonso Filho A, Salles C. Prevalence of insomnia in shift workers: a systematic review. Sleep Sci. 2021;14(1):47–54. 34104337 10.5935/1984-0063.20190150 PMC8157778

[11] Pallesen S , Bjorvatn B, Waage S, Harris A, Sagoe D. Prevalence of shift work disorder: a systematic review and meta-analysis. Front Psychol. 2021;12:638252. doi: 10.3389/fpsyg.2021.638252 33833721 PMC8021760

[12] McIntosh E , Ferguson SA, Dorrian J, Coates AM, Leung G, Gupta CC. “Mars bar and a tin of red bull kept me and my patients alive”: Exploring barriers to healthy eating through facebook comments of shiftworkers. Nutrients. 2023;15(4):959. doi: 10.3390/nu15040959 36839319 PMC9959479

[13] Fauzi MFM , Yusoff HM, Manf MRA, et al. Intervention for occupational fatigue and sleepiness among healthcare workers working in shift: a systematic review. MJPHM. 2019;19(2):47–53.

[14] Reynolds AC , Sweetman A, Crowther ME, et al. Is cognitive behavioral therapy for insomnia (CBTi) efficacious for treating insomnia symptoms in shift workers? A systematic review and meta-analysis. Sleep Med Rev. 2023;67:101716. doi: 10.1016/j.smrv.2022.101716 36459948

